# Protocol for Take-home naloxone In Multicentre Emergency (TIME) settings: feasibility study

**DOI:** 10.1186/s40814-020-00626-w

**Published:** 2020-07-09

**Authors:** Matthew Jones, Fiona Bell, Jonathan Benger, Sarah Black, Penny Buykx, Simon Dixon, Tim Driscoll, Bridie Evans, Adrian Edwards, Gordon Fuller, Steve Goodacre, Rebecca Hoskins, Jane Hughes, Ann John, Jenna Jones, Chris Moore, Fiona Sampson, Alan Watkins, Helen Snooks

**Affiliations:** 1 Swansea University, Wales, UK; 2 Yorkshire Ambulance Service, England, UK; 3 University of the West of England, England, UK; 4 South Western Ambulance Services NHS Foundation Trust, England, UK; 5 University of Sheffield, England, UK; 6 Cardiff University, Wales, UK

## Abstract

**Background:**

Opioids, such as heroin, kill more people worldwide by overdose than any other type of drug, and death rates associated with opioid poisoning in the UK are at record levels (World Drug Report 2018 [Internet]. [cited 2019 Nov 19]. Available from: http://www.unodc.org/wdr2018/; Deaths related to drug poisoning in England and Wales - Office for National Statistics [Internet]. [cited 2019 Nov 19]. Available from: https://www.ons.gov.uk/peoplepopulationandcommunity/birthsdeathsandmarriages/deaths/bulletins/deathsrelatedtodrugpoisoninginenglandandwales/2018registrations). Naloxone is an opioid antagonist which can be distributed in ‘kits’ for administration by witnesses in an overdose emergency. This intervention is known as take-home naloxone (THN). We know that THN can save lives on an individual level, but there is currently limited evidence about the effectiveness of THN distribution on an aggregate level, in specialist drug service settings or in emergency service settings. Notably, we do not know whether THN kits reduce deaths from opioid overdose in at-risk populations, if there are unforeseen harms associated with THN distribution or if THN is cost-effective. In order to address this research gap, we aim to determine the feasibility of a fully powered cluster randomised controlled trial (RCT) of THN distribution in emergency settings.

**Methods:**

We will carry out a feasibility study for a RCT of THN distributed in emergency settings at four sites, clustered by Emergency Department (ED) and catchment area within its associated ambulance service. THN is a peer-administered intervention. At two intervention sites, emergency ambulance paramedics and ED clinical staff will distribute THN to adult patients who are at risk of opioid overdose. At two control sites, practice will carry on as usual. We will develop a method of identifying a population to include in an evaluation, comprising people at risk of fatal opioid overdose, who may potentially receive naloxone included in a THN kit.

We will gather anonymised outcomes up to 1 year following a 12-month ‘live’ trial period for patients at risk of death from opioid poisoning. We expect approximately 100 patients at risk of opioid overdose to be in contact with each service during the 1-year recruitment period. Our outcomes will include deaths, emergency admissions, intensive care admissions, and ED attendances. We will collect numbers of eligible patients attended by participating in emergency ambulance paramedics and attending ED, THN kits issued, and NHS resource usage. We will determine whether to progress to a fully powered trial based on pre-specified progression criteria: sign-up of sites (*n* = 4), staff trained (≥ 50%), eligible participants identified (≥ 50%), THN provided to eligible participants (≥ 50%), people at risk of death from opioid overdose identified for inclusion in follow-up (≥ 75% of overdose deaths), outcomes retrieved for high-risk individuals (≥ 75%), and adverse event rate (< 10% difference between study arms).

**Discussion:**

This feasibility study is the first randomised, methodologically robust investigation of THN distribution in emergency settings. The study addresses an evidence gap related to the effectiveness of THN distribution in emergency settings. As this study is being carried out in emergency settings, obtaining informed consent on behalf of participants is not feasible. We therefore employ novel methods for identifying participants and capturing follow-up data, with effectiveness dependent on the quality of the available routine data.

**Trial registration:**

ISRCTN13232859 (Registered 16/02/2018)

## Background

Accidental overdose related to the misuse of opioid drugs is an increasingly prevalent public health problem, and opioid-related deaths are at record levels in both the UK and North America [1, 2].

People who misuse either illicit or prescription opioids are at an increased risk of non-fatal overdose, subsequent hospital or emergency service utilisation, and death [3–5]. Non-fatal opioid overdose is associated with long-term morbidity and increased demand on health services [6, 7]. Emergency service contact for drug-related morbidity has been found to be a predictor of future episodes of poisoning or overdose [8, 9].

Naloxone is an opioid antagonist used to treat opioid overdose. Naloxone can be supplied to people at risk of opioid overdose by paramedics [10] or by laypeople in the form of take-home naloxone (THN). Typically, a THN kit comprises one or more doses of naloxone, an intramuscular needle and syringe for administering the dose, and written or pictorial instructions to explain how to prepare and administer the dose, perform basic life support, and the importance of calling the emergency services. These materials may also describe the duration of effect and hence why it is important that paramedics attend the patient as soon as possible, the safety of naloxone in terms of adverse events and overdose, and the legality of bystander administration of naloxone.

Non-experimental studies suggest that THN programmes which involve the training of laypersons to administer a naloxone dose in cases of overdose emergency are safe and effective [11, 12]. THN kits can be used by people without formal medical training in the event of an opioid overdose. Increased access to THN kits via specialist drug services in the UK and internationally has been motivated by recommendations from influential bodies, including the World Health Organization (WHO) and the British Advisory Council on the Misuse of Drugs (ACMD) [13, 14].

Numerous THN distribution programmes aiming to reduce death from opioid overdose have been implemented by drug service providers in the UK and internationally since the 1990s [15, 16]. However, a significant proportion of people at risk of opioid overdose do not engage with these services [17]. Additionally, high-quality empirical evidence to demonstrate the safety and effectiveness of THN is sparse. Observational data suggests that non-serious adverse reactions to naloxone administration are common while serious adverse reactions are rare [18]. However, the risks of inadequate response or return to a state of overdose following the administration of naloxone by laypeople remain poorly quantified [19, 20]. Moreover, the uptake of THN kits in at-risk populations remains low [21, 22] and appropriate THN intervention by peers and witnesses may not be optimal [23].

Members of the research team (CM, HS) have previously conducted a randomised feasibility study of THN distributed through the emergency ambulance service in a single-urban geographic area [24]. Their experiences, consistent with those of other researchers [25], have demonstrated that using traditional methods (e.g., telephone or postal methods) for capturing follow-up outcomes of participants in receipt of a THN kit (and of those not in receipt of a THN kit despite eligibility) is not feasible.

This paper describes a protocol for a feasibility study of THN distributed in the Emergency Department (ED) and catchment area within its associated emergency ambulance service. In carrying out the proposed study, we seek to determine the feasibility of carrying out a fully powered cluster randomised controlled trial (RCT) of THN in emergency settings using routinely collected, anonymised, and linked data to capture outcomes. Should we find that carrying out a full trial is feasible, this subsequent trial would be adequately powered to determine the safety, clinical, and cost-effectiveness of THN distribution in emergency settings.

## Methods

### Setting, recruitment, and consent

We will carry out a feasibility study in the emergency care environment, involving study sites defined geographically as an ED and its catchment area within the local emergency ambulance service. For example, one study site comprises the ED at Bristol Royal Infirmary, a large city centre hospital in England, and its catchment area, the surrounding urban area from which the South West Ambulance Service Foundation Trust routinely conveys patients to this ED. The study will be delivered in the form of an RCT clustered by study site, using retrospective anonymised linked routine data to capture patient outcomes. We will also collect qualitative data to gain an understanding of the processes of implementation of the intervention and experiences of service users and providers. Finally, we will collect data related to patient safety.

### Participant recruitment

We cannot know if the naloxone dose included in any individual THN kit will be administered to a peer of the recipient of the kit or to the recipient him/herself. Effects of the THN intervention could extend beyond recipients seen in the ED or by ambulance crews. Therefore, in order to measure treatment effect in those likely to benefit from THN, we define two populations: those eligible for receipt of intervention (at intervention sites) and those at high risk of death from opioid overdose at all sites.

### Population A: eligible for receipt of THN (at intervention sites)

Our first population is the target population for the intervention, comprising adult patients who arrive at the ED, or who are attended by ambulance paramedics for a problem related to opioid misuse (e.g. opioid overdose or injuries due to opioid use). ED clinicians and ambulance service paramedics will undertake an initial clinical assessment as per routine practice, and adults presenting with an opioid misuse-related problem and with the capacity to consent to receipt of the kit will be identified as potentially eligible to receive THN. These patients will be eligible to be offered THN following standard treatment by a participating paramedic or ED clinician.

Patients who lack capacity, who are aggressive or exhibit other challenging behaviour, who are seen by untrained staff, who have already been recruited, or who are in custody at the time of their presentation will be excluded.

### Population B: predicted to be at high risk of death from opioid overdose

We will identify for inclusion in outcome follow-up people at high risk of fatal opioid overdose who may be able to benefit from naloxone from a THN kit. Population B thus extends the target population for follow-up of outcomes beyond original recipients of THN kits. We will define a discriminant function and use this as a predictive tool, similar to a risk index, incorporating known and routinely recorded predictors of opioid-related events. We will use existing linked data on opioid deaths in Wales, including ED and in-patient data, to select predictors most closely associated with those who died from opioid poisoning, and then use these predictors in our discriminant function to identify participants in the study site areas to be included in the ‘high-risk population’ (population B) for outcome analyses. We previously carried out scoping of NHS Wales ED and hospital routine datasets and linked ONS mortality records with these datasets. We found that we were able to describe circumstances of death for opioid overdose decedents who had visited EDs prior to their death, as well as describe service usage over a prolonged observation period. Mortality data were of high quality, as were data items on times and dates of attendances, outcomes of attendances, and demographic characteristics of attendees. Diagnostic and treatment data were of lower quality. Routine data in England will be captured from existing routine datasets as well as the recently implemented ECDS (Emergency Care Data Set); we will assess the quality of the data obtained from this source as a part of the research.

As ambulance service records are not routinely included in nationally available datasets that can be linked anonymously, we will assess whether the inclusion of potential participants from routine ambulance service data, e.g. patient records including flags such as ‘naloxone administered’ or ‘drug overdose’ improves the performance of the predictive tool. If this is the case, these data will be included in the final dataset used to identify the population in whom outcomes will be compared between intervention and control sites.

We will gather prospective clinical data at each intervention site related to eligibility and distribution of THN kits. We will also gather retrospective and prospective data from all participating ambulance services related to indicators of the high risk of death from opioid overdose, e.g. naloxone administration (for use in defining population B). These data will be sent by participating ambulance services and EDs using ‘split-file’ format to the National Health Service (NHS) Digital in England and to the NHS Wales Informatics Service (NWIS) in Wales (with identifiable data separated from clinical data) for matching and linkage to routine centrally held datasets. All study data will be transferred to the Secure Anonymised Information Linkage (SAIL) Gateway for analysis [26].

### Consent

We will not attempt to gain consent to participate in the trial prospectively, at the time of attendance for opioid-related emergency, because that setting contradicts the requirements of informed consent [27]. We will not gather consent retrospectively, as the population is likely to be very difficult to reach and low contact rates could invalidate research findings. We will, however, consent patients to receive the intervention. Patients will do this by signing a training sheet, giving their name and date of birth as part of this process. In this way, we will gather some demographic information about the trainees and have a written record confirming that participants were trained to use the THN kit effectively.

As the wider population for inclusion in follow-up (population B) will be identified through anonymised routine data sources, we will not have identifiable data with which to contact people for consent purposes. Rather, we will offer the option to dissent from the research at all sites via patient information leaflets supplied with THN kits and made available at ED waiting areas. We will also include this information on the Wales Centre for Primary and Emergency (including unscheduled) Care Research (PRIME) website www.primecentre.wales. We have gained ethical, research, and information governance permissions to allow this study to follow this approach, in which all information about processes and outcomes of care will be anonymised to the research team except for clinical members at each site. These clinical researchers will split identifiable from clinical and operational study data before sending files separately to the NHS Digital in England and NWIS in Wales for linkage to routinely held outcomes in ED, in-patient, and mortality datasets held centrally; this split-file approach in which identifiable and clinical data are separated preserves patient anonymity [25].

For the qualitative component, we will obtain written informed consent from all service users and health care professionals who participate in interviews and focus groups. Service user participants will be identified by members of the NHS care team and third-sector drug treatment services. Participants will also be eligible to receive a thank you gift card voucher for their time.

### Sample size

**Fig. 1:**
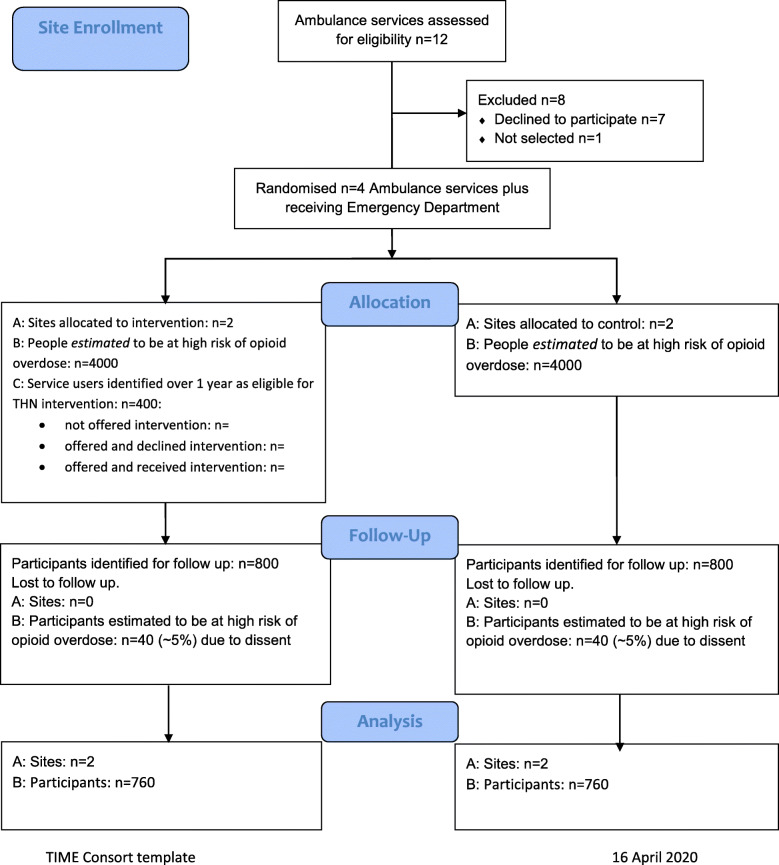
Overall CONSORT diagram

We aim to include enough patients to test our study methods, study intervention, and outcome data collection. Site enrolment and allocation, and follow-up of participants prior to analysis are summarised in Fig. 1.

At each intervention site, with approximately 2000 people thought to be at high risk of fatal overdose, we expect 200 to make contact with the ED or ambulance service over the course of 1 year and be eligible to receive THN. We expect 100 contacts via the ED, and another 100 contacts via the corresponding ambulance service.

We will use routine linked data to identify the population to be included at each site in outcome analyses via a predictive model to be fully specified within our study. The model will include opioid users who have made contact with included services over a period of 24 months previous to our recruitment phase. Individuals predicted to be at risk of death from opioid overdose will include those attending ED or being attended by a 999 paramedic during the recruitment phase and their peers. We expect the follow-up population to be at least 1520 people across four sites.

#### Randomisation

We approached all UK ambulance services and received five positive responses from potential sites with matched EDs, who were able to demonstrate the capacity and resources to participate. Of these potential sites, four demonstrated sufficient geographic separation from other study sites to mitigate potential cross-contamination of study populations. From these four sites, we selected two to be intervention sites and two to be control sites; a member of the research team (MJ) picked one set of study site allocations at random from the set of all possible allocations, each contained within separate sealed opaque envelopes.

#### Intervention

The TIME intervention, described here according to guidance for intervention description and replication (TIDieR) checklist [28], is Prenoxad—a multi-dose THN kit containing 2mg naloxone hydrochloride 1mg/1ml solution for intramuscular injection. This kit contains simple textual and pictorial instructions which reiterate face-to-face training each participant receives as part of the intervention. Participants will not be able to receive part of the intervention only—e.g. the training but not the kit—and so will need to consent to the whole intervention or decline the whole intervention.

The kit is manufactured by the Martindale Pharma (Woodburn Green, UK) and supported by ‘train-the-trainer’ materials for participating paramedics and ED staff developed by Stephen Malloy, an independent consultant. Each Prenoxad kit retails at £21.00 before VAT (value-added tax). The decision to use the Prenoxad kit, as opposed to an intranasal alternative, is supported by evidence regarding the bioavailability of naloxone following intramuscular versus intranasal administration [29, 30] and the time taken for improvement in respiratory rate to be observable [31]. We also based our decision on feedback from drug service workers who were approached in the initial setting up of the study.

Paramedics, nurses, and doctors at intervention site EDs and ambulance services registered with their respective professional bodies will be invited to participate in the study, and volunteers will be trained in delivering the intervention in accordance with the study protocol. Patient Group Directions (PGDs) will be established at participating services within intervention sites to allow non-prescribing paramedics and nurses to distribute THN kits. Training, provided in a flexible manner to suit the working practices of individual departments and services, will involve face-to-face group-based training, complemented by a ‘cascade’ approach whereby research support paramedics and nurses continue to train their peers on an ad hoc basis. Online resources produced by Martindale Pharma will be available as refresher content for staff (http://www.prenoxadinjection.com/). Training per person will take up to 15 min. Staff will complete and sign a ‘Record of Completion of Training’ form once they have been deemed competent by their trainer.

At intervention sites, participating healthcare professionals based in the specific ED or ambulance service region and caring for patients eligible to receive the intervention will offer these patients the THN kit, with an explanation of its purpose. Patients will receive treatment as usual (TAU) and then be offered the intervention.

TAU for suspected opioid overdose involves a clinical assessment during which the healthcare staff who first come into contact with the patient seek to confirm the substance or substances which led to the overdose. Opioid overdose is assumed if the substance which led to overdose is not known and the patient presents with an altered mental state—including reduced consciousness—bradypnoea and miosis. Treatment includes prolonged and gradual administration of naloxone followed by a period of observation. Ideally, for patients attended by ambulance services, the treatment will begin at the site of the overdose and then continues at the ED following conveyance. However, patients may refuse to be conveyed should they respond to the naloxone at the scene.

In the intervention arm, if the patient consents to receiving the kit, the healthcare professional will provide training regarding the preparation and administration of the naloxone dose using the kit materials. The healthcare professional and the patient will then complete a training checklist document, to be made available to research support staff and stored as evidence that training was provided as part of the intervention.

At control sites, patients who attend for opioid poisoning or overdose or other drug-related problems will receive TAU (as described above) from ambulance and ED staff and will not be offered a THN kit.

#### Blinding

Due to the nature of the intervention, the study will not include blinding of participants or intervention providers. However, the study statistician will remain blinded to allocation until the study data is ‘locked’ and as far as proves feasible during analysis.

### Outcomes

We will measure outcomes related to the feasibility of the study in terms of the intervention and methodology, including whether we can capture sufficient data to measure clinical outcomes and health economics.

Outcomes related to the feasibility of the study include sign up of sites; proportion of eligible staff recruited and trained to deliver the intervention; proportion of eligible participants identified, number of kits issued; and the adverse event rate in intervention and control sites.

We will also assess the feasibility of collecting clinical outcomes from anonymised linked routine health records over a period of 12 months, acknowledging that feasibility will also depend on the quality and availability of routine data.

The proposed primary outcome is mortality (all deaths and those known to be opioid-related). Secondary outcomes include intensive treatment unit (ITU) admissions, ED visits, and in-patient admissions (all visits/attendances as well as those known to be opioid-related), further 999 calls as well as THN kits issued and costs. Our feasibility study will not be adequately powered to detect statistically significant differences in these proposed outcomes between intervention and control arms.

We will assess the feasibility of using routine data sources to estimate health care costs. Total NHS costs for each patient will be calculated based on the staff training costs, patient training costs, and other NHS costs (e.g. those for 999 calls, ED attendances, and admissions). Training costs will be calculated using records of completion of training, staff recall of patient training, and then combined with NHS salary data. Other NHS costs will be based on routine data for the relevant ED and ambulance service trusts.

We will use qualitative data to explore the feasibility and acceptability of THN from the perspective of service users, based upon their previous knowledge and experience of overdose. We will also explore the feasibility and acceptability of the intervention from the provider perspective by undertaking interviews and focus groups with paramedics, clinical ED staff, and health service managers at participating sites regarding THN in emergency settings. We will explore awareness and experiences of naloxone, perceived benefits and challenges of THN, and views on the feasibility and acceptability of distributing THN via ambulance paramedics and hospital EDs. Interviews will be recorded, with participants’ consent, and professionally transcribed prior to analysis. We will use normalisation process theory (NPT) to guide analysis of the provider data and to help understand how the intervention can be optimised within the ED and prehospital settings, and to explore whether difficulties in implementation are due to the intervention itself or due to other factors.

### Progression criteria

We will decide whether or not to proceed to a fully powered RCT using the following assessment principles and progression criteria:

- Green: indicates that we have either met a criterion (in which case no modifications to the relevant aspect of the study protocol may be needed) or we are within 10% of our stated progression targets (in which case we will review the reasons for this and consider appropriate modifications to study methods)
- Amber: indicates that we are within 20% of our stated progression target, in which case we will critically review reasons for this and assess whether major changes to study methods are likely to realise significant improvements
- Red: indicates that we are more than 20% from our target, in which case we will not, in the absence of clear extenuating circumstances, consider progression to a full trial

All the percentage changes will be measured as relative.

### Intervention feasibility

1. Sign up of four sites, including ≥ 50% eligible staff to complete training in delivering the intervention at each intervention site
2. Identification of ≥ 50% of people who have presented to ED or ambulance service with opioid overdose or an opioid use related problem
3. THN kits offered to ≥ 50% eligible patients at intervention sites
4. Serious adverse event rate of no more than 10% difference between intervention and control sites during the live trial period and prior to the conclusion of data collection

### Trial method feasibility

1. Identification and inclusion for outcome follow-up of ≥ 75% of people who died of opioid poisoning in the following year
2. Matching and data linkage in ≥ 90% of cases not dissented at the conclusion of quantitative data collection
3. Retrieval of primary and secondary outcomes for ≥ 75% of included participants from NHS Digital and National Welsh Informatics Service within 1 year of the projected timeline

### Safety monitoring

We will regard data on service usage (emergency and ITU admissions, and emergency attendances) and death as surrogate markers of adverse behavioural change in relation to opioid misuse, such as taking larger doses at one time. In doing so, we assume that increased rates of service usage and death correlate with an increased volume of higher risk drug-taking behaviour. We will also monitor for instances of serious adverse events, including deaths following THN use by interrogating routine health service data and also requests for data made to services on behalf of coroners across intervention and control sites. We include control sites because we expect THN to be available from specialist drug services in control sites.

### Data analysis

#### Discriminant function

To develop the discriminant function to be used for defining our population for outcome comparison, we will partition the available routine (retrospective) linked dataset, using one part (training data) to determine inclusion thresholds and the other (testing data) to check the performance of the function. We will use the actual number of recorded opioid poisoning deaths during the trial period to validate the function. We will summarise performance by calculating sensitivity (the proportion of actual opioid poisoning deaths included in our defined high-risk population; denominator = all actual opioid poisoning deaths, a/a+c) and positive predictive value (the proportion of our defined the high-risk population who die of opioid poisoning in the following year; denominator = all defined high risk, a/a+b) as shown in Table 1.

**Table 1:** Sensitivity and positive predictive value of a discriminant function

	Actual opioid death	No opioid death	
Predicted high risk	a	b	a+b
Not predicted high risk	c	d	c+d
	a+c	b+d	

We will report on whether our discriminant function can, via Fisher’s linear discriminant function, be usefully reduced to a single individual-level risk score, consider thresholds used in its definition, and evaluate its performance as a predictive tool using test datasets compiled specifically for this purpose.

#### Study data

Our primary analyses will address the progression criteria as presented above and will be largely descriptive in nature. We will develop a formal Statistical Analysis Plan (SAP) to outline all planned analyses, including conventions on the treatment of missing data, principles of selection of explanatory factors and covariates in statistical models, and the reporting of raw and adjusted outcomes.

We will produce a CONsolidated Standards Of Reporting Trials (CONSORT) flowchart for patient recruitment appropriate for cluster trials [32]. Study data will be summarised by intervention or control arm, and we will further summarise key demographic and outcome variables by study site. Although this feasibility study is not intended to provide a definitive evaluation of the THN intervention, we will assess and report differences in outcomes via appropriate generalised mixed linear models, adjusting for key covariates and factors.

For the follow-up population (B), we will also summarise linkage rates and characteristics for those not linked versus those linked, coding completion rates for ED and ambulance service events, and carry out a comparison of data obtained from routine sources. We will report these data by site and in total. We will provide details on data completeness related to the criteria for determining progression to a full trial.

#### Qualitative data

We will use NVIVO to manage the qualitative data and carry out a thematic analysis of interview transcripts. Transcripts will be imported into NVIVO and be read and re-read to ensure familiarity with the data before coding. One researcher will develop and refine initial codes, with coding undertaken both with reference to NPT as a theory for evaluating the potential for normalisation of the intervention as a change of working practices in emergency settings [33] and ‘grounded’ in the data. Codes will be reviewed and emerging concepts used to develop themes. A second researcher will independently code a subsample of transcripts for comparison and discussion before further refinement of the coding structure and themes by the first researcher. Patient and public involvement (PPI) members will be involved in the qualitative analysis by co-developing themes and reviewing drafts of findings. Data from service providers at intervention sites will be compared within sites across time and also between sites for commonalities and divergence in themes.

### Trial management

A Trial Management Group (TMG) will manage the project and report to the independent Trial Steering Committee (TSC) at appropriate intervals. The Chief Investigator will chair the TMG which will meet every 3 months. The TMG will comprise all co-applicants, named collaborators, PPI members, and researchers.

The TSC will oversee the conduct and progress of the trial and adherence to the protocol, patient safety, and the consideration of new information of relevance to the trial. Two PPI members are full members of the TSC.

A Data Monitoring Committee (DMC) will monitor study data at interim periods and make recommendations to the TSC on whether there are any ethical or safety reasons why the trial should not continue. Its members will have access to comparative data and interim analyses and may request the un-blinding of such data at any time. The DMC will also consider requests for the release of data. The DMC may be asked by the TSC, Trial Sponsor, or Study Funder to consider data emerging from other related studies. If new evidence becomes available during the course of the trial, it is the responsibility of the trial and/or Data Manager to provide that information to the DMC to allow them to consider such issues and make recommendations on the continuation of the trial to the TSC.

Any risks identified throughout the trial will be documented in a risk log and monitored. This will be reported to the TMG and escalated to the TSC if appropriate.

### Public and patient involvement

We have involved public and patient members throughout this study to strengthen research rigour [34]. PPI members have experience of opioid addiction through family and voluntary networks. They have contributed to developing this study using personal experience to highlight the relevance of the research questions and comment on data collection methods and selection of outcomes. We also discussed the project with drug service users and voluntary sector service providers in the community. PPI members were named as co-applicants on the funding proposal. They will remain involved as members of the Trial Management Group and relevant subgroups. They will contribute to study management, reporting and dissemination through papers. Additionally, we have recruited two more individuals with relevant experience to be involved in the Trial Steering Committee. We will support all public and patient members to collaborate as equal members throughout the study [35].

#### Dissemination

In addition to publishing results in scientific journals, we will engage with third sector organisations, and media and communication departments at participating institutions, and we will disseminate findings and raise awareness about the trial and wider issues related to implementation. We will develop a proposal for funding for a fully powered trial, should this be supported by our findings.

## D**iscussion**

This study is the first to our knowledge to use routine anonymised linked data to identify the target population and measure outcomes related to a peer administered anti-overdose intervention such as THN in emergency settings. Our novel approach to capturing outcome data comes from the public health nature of the intervention, which is administered by laypeople to their peers who may or may not be the original recipient of the THN kit, as well as our own previous research indicating that individuals within the target population for the intervention move around a lot and are very difficult to follow-up using traditional methods.

The strength of this study lies in its novel approach establishing evidence in a new setting for an intervention which is already being distributed, albeit patchily, in response to opioid overdose as a growing public health concern. The study is being carried out by a team well placed to apply expertise and prior experience in the use of routine data in RCTs to contribute to the evidence base for THN in emergency settings, which is in urgent need of strengthening.

## Data Availability

Not applicable

## Abbreviations

ACMD: Advisory Council of the Misuse of Drugs
CONSORT: CONsolidated Standards Of Reporting Trials
DMC: Data Monitoring Committee
ECDS: Emergency Care Data Set
ED: Emergency
ITU: Intensive treatment unit
NHS: National Health Service
NPT: Normalisation process theory
NWIS: NHS Wales Informatics Service
PGD: Patient Group Direction
PPI: Patient and public involvement
PRIME: Primary and Emergency (including unscheduled) Care Research
RCT: Randomised controlled trial
SAIL: Secure Anonymised Information Linkage
SAP: Statistical Analysis Plan
THN: Take-home naloxone
TIDieR: Template for Intervention Description and Replication
TIME: Take-home naloxone in multicentre emergency setting
TMG: A Trial Management Group
TSC: Trial Steering Committee
VAT: Value-added tax
WHO: World Health Organization
